# Genome-Wide Genetic Analysis of Dropout in a Controlled Exercise Intervention in Sedentary Adults With Overweight or Obesity and Cardiometabolic Disease

**DOI:** 10.1093/abm/kaae011

**Published:** 2024-03-15

**Authors:** Rong Jiang, Katherine A Collins, Kim M Huffman, Elizabeth R Hauser, Monica J Hubal, Johanna L Johnson, Redford B Williams, Ilene C Siegler, William E Kraus

**Affiliations:** Department of Head and Neck Surgery & Communication Sciences, Duke University School of Medicine, Durham, NC, USA; Duke Molecular Physiology Institute, Duke University School of Medicine, Durham, NC, USA; Department of Population Health Sciences, Duke University School of Medicine, Durham, NC, USA; Duke Molecular Physiology Institute, Duke University School of Medicine, Durham, NC, USA; Department of Medicine, Duke University Medical Center, Durham, NC, USA; Duke Molecular Physiology Institute, Duke University School of Medicine, Durham, NC, USA; Department of Biostatistics, Duke University School of Medicine, Durham, NC, USA; Department of Kinesiology, Indiana University Purdue University Indianapolis, Indianapolis, IN, USA; Duke Molecular Physiology Institute, Duke University School of Medicine, Durham, NC, USA; Department of Psychiatry and Behavioral Sciences, Duke University School of Medicine, Durham, NC, USA; Department of Psychiatry and Behavioral Sciences, Duke University School of Medicine, Durham, NC, USA; Duke Molecular Physiology Institute, Duke University School of Medicine, Durham, NC, USA; Department of Medicine, Duke University Medical Center, Durham, NC, USA

**Keywords:** Gene expression, Metabolism, Energy metabolism, Non-completion

## Abstract

**Background:**

Despite the benefits of exercise, many individuals are unable or unwilling to adopt an exercise intervention.

**Purpose:**

The purpose of this analysis was to identify putative genetic variants associated with dropout from exercise training interventions among individuals in the STRRIDE trials.

**Methods:**

We used a genome-wide association study approach to identify genetic variants in 603 participants initiating a supervised exercise intervention. Exercise intervention dropout occurred when a subject withdrew from further participation in the study or was otherwise lost to follow-up.

**Results:**

Exercise intervention dropout was associated with a cluster of single-nucleotide polymorphisms with the top candidate being rs722069 (T/C, risk allele = C) (unadjusted *p* = 2.2 × 10^−7^, odds ratio = 2.23) contained within a linkage disequilibrium block on chromosome 16. In Genotype-Tissue Expression, rs722069 is an expression quantitative trait locus of the *EARS2*, *COG7*, and *DCTN5* genes in skeletal muscle tissue. In subsets of the STRRIDE genetic cohort with available muscle gene expression (*n* = 37) and metabolic data (*n* = 82), at baseline the C allele was associated with lesser muscle expression of *EARS2* (*p* < .002) and *COG7* (*p* = .074) as well as lesser muscle concentrations of C2- and C3-acylcarnitines (*p* = .026).

**Conclusions:**

Our observations imply that exercise intervention dropout is genetically moderated through alterations in gene expression and metabolic pathways in skeletal muscle. Individual genetic traits may allow the development of a biomarker-based approach for identifying individuals who may benefit from more intensive counseling and other interventions to optimize exercise intervention adoption.

**Clinical Trial information:**

STRRIDE I = NCT00200993; STRRIDE AT/RT = NCT00275145; STRRIDE-PD = NCT00962962.

## Teaser

Exercise intervention dropout appears to be genetically moderated through alterations in gene expression and metabolic pathways in skeletal muscle among sedentary adults with overweight or obesity and cardiometabolic disease.

## Background

There are numerous health benefits of chronic exercise training, including those for cardiovascular disease, diabetes mellitus, cancer, cardiometabolic diseases, brain health, and others [1–3]. However, despite these recognized health benefits, many persons fail to successfully adopt an exercise program when initially motivated. Failure to adopt an exercise program likely results from a combination of social, environmental, behavioral, personality, and biochemical determinants, many of which are likely to be moderated by underlying genetic variants. Thus, while clinicians recommend patients improve their health by increasing physical activity, those most likely to benefit from increasing physical activity may be those who are least likely to adopt an exercise program. Prior work in large-scale twin studies and animal models have identified promising quantitative trait loci associated with physical activity level, with heritability of physical activity in adults ranging from 20% to 90% [4–14]. Although well explored in rodents, this issue is unexplored in human intervention studies. Furthermore, being physically inactive or dropping out of an exercise intervention likely involves different mechanisms regulating these different behaviors, leaving a gap in this research area. In addition, individuals who dropout are generally not studied for the effectiveness of the exercise intervention, widening the gap even further. We hypothesize genetic predisposition to poorer exercise capacity or training responses—at least partly mediated by metabolic pathways in skeletal muscle—are key components of the ability to adopt an exercise intervention.

We previously described several genetic variants in linkage disequilibrium (LD) on the acid ceramidase gene (ASAH) related to dropout from a structured exercise training program, using a candidate gene approach [15]. For those completing the exercise intervention, these variants were associated with a poorer training response in cardiorespiratory fitness ($\dot{V}O_{2peak}$) to any given exercise intervention, and differential gene expression in skeletal muscle, providing a biological construct for how genetic factors can mediate poor exercise behavior [15]. These findings highlight how genetic variations may fundamentally influence an individual’s physiologic experience during exercise. Variability in an individual’s physiologic experience during exercise could be a result of differences in muscle capacity and metabolism, which may lead to a less efficient muscle response, reduced exercise tolerance, and/or greater perceived exertion during exercise. This greater perceived exertion may play a critical role in an individual’s satisfaction with perceived physical benefits, or lack thereof, as well as shaping an individual’s affective response to exercise. This consistent feedback loop of perceived failure to meet physical expectations and adaptations over time can lead to negative emotional responses to exercise, decreased motivation to continue exercise, and increased likelihood of intervention dropout [16–22]. Thus, further investigation into how genetic predispositions may not only determine physical outcomes but also shape the psychological framework within which an individual perceives exercise and is motivated to continue the behavior change process is critical.

While our previous approach has successfully identified genes associated with exercise response, targeted genetic studies may miss novel biologically significant polymorphisms. Therefore, in this report, we undertook a genome-wide association study (GWAS) approach to identify other putative genetic variants associated with dropout from exercise training interventions among 603 individuals in the STRRIDE (Studies of a Targeted Risk Reduction Intervention through Defined Exercise) randomized trials. We hypothesized any putative, GWAS-identified genetic variants associated with dropout would be associated with differential baseline and/or exercise-induced changes in physiologic parameters associated with energy metabolism (e.g., $\dot{V}O_{2peak}$; skeletal muscle gene expression and metabolites)—thereby, conferring biological plausibility and putative mechanism for the findings. Understanding the genetic determinants of exercise behavior will be important for implementing guidelines for physical activity and health, as well as for targeting interventions for those at a greater risk of dropout from their initiated exercise intervention or program.

## Methods

### Study Population

**Fig. 1.**
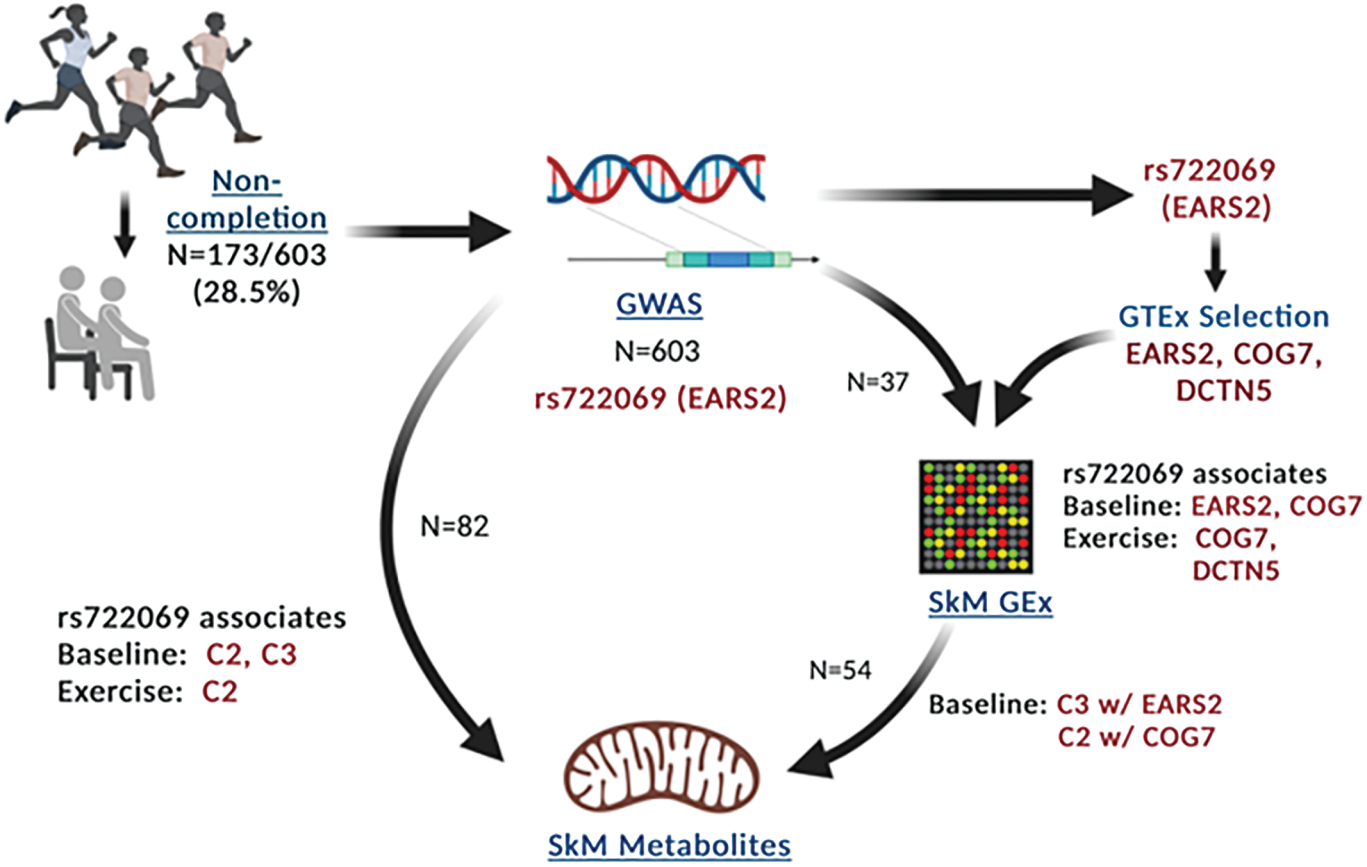
Study design for the genome-wide association study (GWAS) and genomic analysis of intervention dropout in the STRRIDE exercise intervention studies. *Exercise* post-exercise training; *GEx* gene expression; *GTEx* genotype-tissue expression; *SkM* skeletal muscle.

Data and biological samples (DNA and muscle biopsies) were available for 603 individuals from three independent, controlled exercise trials: STRRIDE I (NCT00200993), STRRIDE AT/RT (NCT00275145), and STRRIDE-PD (NCT00962962), conducted between 1999 and 2012. A complete description of the STRRIDE I, AT/RT, and PD designs are published elsewhere [23–25]. Participants were: 18–75 years of age; sedentary; with overweight or obesity (body mass index: 25–35 kg/m^2^); and with mild-to-moderate lipid abnormalities or prediabetes. In each study, participants were randomized to different exercise groups, a non-exercising control group in STRRIDE I, and a “diet plus exercise” group modeled after the Diabetes Prevention Program in STRRIDE-PD. Those participants with DNA available for genetics studies from all three STRRIDE cohorts were combined and analyzed in this study. Participants with muscle tissue obtained by biopsy were used for functional genomics analysis. A conceptual diagram for the design of this study is shown in Fig. 1 and sample sizes for each analysis are shown in Supplementary Fig. S1. All participants provided verbal and written informed consent as approved by the Duke University Institutional Review Board and ECU Institutional Review Board.

### Exercise Training

The detailed exercise interventions for each STRRIDE cohort are described in the Supplementary Materials. Each of the exercise interventions was characterized in terms of amount, intensity, mode, and inclusion of a dietary component. To allow comparison across the studies, intervention groups were recoded according to these features as shown in Supplementary Table S1. After randomization to one of these groups, participants began the exercise intervention phase. After randomization, dropout was defined as withdrawal from the study for any of the following reasons: time constraints; injury or illness unrelated to the study; medical problems; family issues; or geographic relocation and others. There was no differential distribution for these reasons across exercise groups for each of the three studies. For the purposes of the GWAS statistical analysis, dropout was defined as a participant who withdrew after randomization and a completer was defined as a participant who continued to the end of the study, regardless of compliance with the assigned exercise intervention.

### GWAS SNP Genotyping and Sample Quality Control

DNA was isolated from whole blood using a commercial DNA isolation kit and a standard protocol (Qiagen, Inc, Valencia, CA). Genome-wide single-nucleotide polymorphism (SNP) genotypes were assayed using Illumina OmniExpressExome-8 v1.4, and called using Illumina’s GenomeStudio software. A sequence of filtering steps based on the metrics available in GenomeStudio was applied to remove problematic SNPs, including: removing markers with AB T mean <0.2 or ≥0.8; removing markers with cluster separation <0.3, removing markers with het excess ≥0.2 or het excess ≥0.3; removing markers with >3 called genotype clusters; removing any remaining SNPs with >3 replication errors; and removing all remaining markers with call rate <98%. We also checked for batch genotyping effects by performing a chi-square test for differential allele frequencies per marker across the three groups. Sixteen samples were genotyped in duplicate with 99.9% reproducibility across all the SNPs (data not shown). The genotyping calls were output as a PLINK data file and further quality control was performed in the following steps: removing samples with sex mismatches (predicted compared with the actual sex); removing samples with SNP call rate <98%; removing outliers by plotting heterozygosity rate versus proportion of missing genotypes; and checking relatedness by removing one sample in the pairs with lowest SNP rate if inheritance-by-descent estimation >0.2. All SNPs were filtered by minor allele frequency >0.05, and Hardy–Weinberg equilibrium test (*p* > .001). A principal component analysis was performed to allow adjustment for population stratification (principal components [PCs], Supplementary Figs. S2 and S3). There were 16 samples removed during quality control and 36 samples with missing exercise training information, indicating the subjects dropped out during run-in testing and before group randomization. After quality control, a total of 603 post-randomization samples with genotype data at 584,117 variants were available for GWAS analysis. Population stratification was assessed with a *Q*–*Q* plot of the *p*-values (Supplementary Fig. S4) with no evidence of deviation from the assumed distribution.

### Skeletal Muscle Metabolites

Given our underlying hypothesis focused on metabolic mediators of training responses and their correlates with dropout genes, skeletal muscle metabolite data were collected in randomly chosen 120 training completers from STRRIDE I and AT/RT equally distributed among five exercise groups and controls—20 in each group (Supplementary Fig. S1) [26]. Before and at the conclusion of the intervention training (16–24 hr after the last exercise bout), *vastus lateralis* needle biopsies were performed in fasted participants using a modified Bergstrom technique [27]. Samples were snap frozen and stored at −80°C. Muscle tissue was diluted 20 times (mass:volume) in ice-cold water, minced with surgical scissors and homogenized for 30 s using a rotor–stator tissue disruptor. A targeted, mass-spectrometry-based platform was used to measure a total of 15 amino acids, 45 acylcarnitines, and 7 organic acids as previously described [28, 29]. For analyses of amino acids and acylcarnitines, samples were precipitated with methanol, esterified and analyzed with tandem mass spectrometry (MS/MS). Organic acids were measured with gas chromatography–mass spectrometry (GC/MS) [28]. All participants’ longitudinal samples were prepared and analyzed in a single batch. Three batches of participants’ samples were prepared and analyzed.

### Skeletal Muscle Gene Expression Profiling

We conducted pre- and post-training skeletal muscle gene expression analysis on a subset of 60 participants having skeletal muscle metabolite data as above [15]. The gene expression subgroup included ten subjects (five men and five women) from each of six exercise groups from STRRIDE I and AT/RT; paired baseline and post-training samples were always processed identically in the same assay run. Total RNA was extracted using the standard Trizol (Invitrogen, Carlsbad, CA) method and 30–50 mg of starting skeletal muscle. Two-round amplification of total RNA was performed using a commercially available kit (Affymetrix, Santa Clara, CA). Thirty micrograms of biotinylated cRNA from each sample were hybridized to Affymetrix U133 Plus 2.0 microarrays. More detailed methods associated with microarray gene expression analysis can be found elsewhere [30, 31]. Probe set expression levels were generated using the PLIER algorithm (typically six iterations) in Expression Console (Affymetrix) and imported directly into Partek Genomics Suite (Partek Inc., St. Louis, MO) for statistical processing. Probe set intensities (PLIER) were log_2_-transformed for gene expression data normalization. Of those with metabolite data, a total of 56 samples met all quality control metrics for analysis of skeletal muscle transcript concentrations. Standard quality control measures for adequate amplifications, thresholds for appropriate scaling factors, and RNA integrity (GAPDH 3_/5_ and HSAC07 3_/5_) were assessed. Samples not meeting quality control standards were reprocessed from the original total RNA after RNA integrity was verified by agarose gel electrophoresis and imagining [32].

### Statistical Analysis

#### GWAS of dropout

GWAS analyses were performed using the PLINK software in Linux (version 1.9, www.cog-genomics.org/plink/1.9/) [33]. Additional analyses were performed using SAS (version 9.4, SAS Inc, Cary, NC) or R (version 4.0.1, https//www.r-project.org). Dropout was coded as a binary outcome according to whether the participant completed the study (completed = 0, non-completed = 1). Logistic regression modeling was conducted for dropout associated with each SNP, adjusted for age, gender, intervention code (diet, resistance, amount, and intensity), study cohort (STRRIDE I = 1, STRRIDE AT/RT = 2, STRRIDE-PD = 3), and PCs. SNPs were modeled as an additive effect with 0, 1, or 2 copies of the minor allele. By convention, conservative genome-wide and suggestive statistical significance were defined as *p* < 5 × 10^−8^ and *p* < 10^−4^, respectively. The coefficient and *p*-value for each SNP were ranked and those SNPs with the smallest *p*-values were evaluated for further association analysis with expression and metabolite levels. We evaluated the replication of top SNPs in published GWAS for seven exercise traits related to intensity or amount using the Atlas of GWAS Summary Statistics (https://atlas.ctglab.nl/) [34].

#### Genetic effects on gene expression in skeletal muscle

SNPs with the smallest *p*-value (*p* < 10^−5^) from the GWAS analysis were evaluated for expression differences by genotype as expression quantitative trait loci (eQTL) in three ways First, we conducted an in silico search in GTEx Portal (Genotype-Tissue Expression; https://gtexportal.org/home/, a comprehensive public resource to study tissue-specific gene expression and regulation) for an association between genotype and expression level in skeletal muscle tissues, to identify genes potentially regulated by the top GWAS SNPs. The GTEx Project was supported by the Common Fund of the Office of the Director of the National Institutes of Health, and by NCI, NHGRI, NHLBI, NIDA, NIMH, and NINDS. The data used for the analyses described in this manuscript were obtained from: the GTEx Portal on 07/05/2019 and/or dbGaP accession number phs000424.v9.p2. We examined skeletal muscle eQTLs to prioritize candidate SNPs within genomic regions. Second, we examined the association among SNPs and skeletal muscle gene expression concentrations before intervention (pre-expression) using linear regression models controlling for age, gender, race, and STRRIDE cohort (equation 1); these analyses were performed in a subset of the sample (*n* = 37) with skeletal muscle gene expression data available. Third, the same model (equation 2) as in step 2 was evaluated for gene expression concentrations after intervention (post-expression) with the inclusion of the gene expression concentrations before intervention (pre-expression) is included as a covariate.


$$\begin{array}{l} \begin{array}{lll} Pre\text{-}expression= & \;\;\beta_{0}+\beta_{1}SNP+\beta_{2}age+\beta_{3}gender+\; & \beta_{4}race+\beta_{5}cohort+\;\varepsilon \; \end{array} \end{array}$$ (1)

$$\begin{array}{l} \begin{array}{lll} Post\text{-}expression= & \;\;\beta_{0}+\beta_{1}SNP+\beta_{2}age+\beta_{3}gender+\beta_{4}race+\; & \beta_{5}cohort+\beta_{5}pre\text{-}expression+\;\varepsilon \; \end{array} \end{array}$$ (2)

#### Genetic effects on metabolites in skeletal muscle

A subset (*n* = 82) was used to examine the associations among top SNP effects and skeletal muscle metabolite concentrations (log_2_-transformed). Similar linear regression models were performed separately for pre-training metabolites (equation 3) and post-training metabolites (equation 4), adjusting for batch effects from the metabolite measurements.


$$\begin{array}{l} \begin{array}{lll} Pre\text{-}metabolite= & \;\;\beta_{0}+\beta_{1}SNP+\beta_{2}age+\beta_{3}gender+\beta_{4}race+\; & \beta_{5}batch+\beta_{6}cohort+\;\varepsilon \; \end{array} \end{array}$$ (3)

$$\begin{array}{lll} Post\text{-}metabolite= & \;\;\beta_{0}+\beta_{1}SNP+\beta_{2}age+\beta_{3}gender+\beta_{4}race+\; & \beta_{5}batch+\beta_{6}cohort+\beta_{7}pre\text{-}metabolite+\;\varepsilon \; \end{array}$$ (4)

#### Gene expression associated with metabolites in skeletal muscle

The associations among muscle gene expression and metabolite concentrations (*n* = 54) were analyzed using linear regression models. Metabolite concentrations were log_2_-transformed prior to statistical testing. To determine whether pre-training metabolite concentrations were related to pre-training gene expression concentrations, pre-training metabolite concentrations were modeled as outcomes with pre-training expression concentrations as the effect of interest, adjusted for age, gender, race, STRRIDE cohort, and metabolite batch effect (equation 5). To examine the effects of pre-training gene expression and changes in gene expression on post-intervention metabolite concentrations, post-metabolite concentrations were modeled as outcomes, and both pre- and post-training expression concentrations were modeled as outcomes, controlling for the same covariates (equation 6). For each of the functional models, we used a *p*-value <.05 to indicate statistical significance.


$$\begin{array}{l} \begin{array}{lll} Pre\text{-}metabolite= & \;\;\beta_{0}+\beta_{1}pre\text{-}expression+\beta_{2}age+\beta_{3}gender+\; & \beta_{4}race+\beta_{5}batch+\beta_{6}cohort+\;\varepsilon \; \end{array} \end{array}$$ (5)

$$\begin{array}{l} \begin{array}{llll} Post\text{-}metabolite= & \;\;\beta_{0}+\beta_{1}pre\text{-}expression+\beta_{2}age+\beta_{3}gender+\; & \beta_{4}race+\beta_{5}batch+\beta_{6}cohort+\; & \beta_{7}post\text{-}expression+\beta_{8}pre\text{-}metabolite+\;\varepsilon \; \end{array} \end{array}$$ (6)

## Results

### Sample Characteristics

Overall, participants were on average 52.9 ± 9.3 years old, European-American (76.6%), women (53.6%), and with obesity (30.4 ± 3.0 kg/m^2^). The characteristics of participants by completers (*n* = 431, 71.5%) and dropouts (*n* = 172, 28.5%) are provided in Table 1. The two groups were largely similar in age, biological sex, STRRIDE cohort, diet, amount, and intensity, while dropouts were more likely to be African-American and randomized to a group that did not include a resistance component (*p* < .05).

**Table 1:** Distribution (*N*, %) of Participants in the STRRIDE Genome-Wide Association Study (GWAS) by Completers and Dropouts

	Completers	Dropouts	Total
Total *N*	431 (71.5)	172 (28.5)	603
Age (years, *SD*)	53.3 (9.1)	52.0 (9.6)	52.9 (9.3)
Body mass index (kg/m^2^, *SD*)	30.2 (3.0)	31.0 (3.1)	30.4 (3.0)
Gender			
Men	209 (48.5)	71 (41.3)	280 (46.4)
Women	222 (51.5)	101 (58.7)	323 (53.6)
Race*			
European-American	350 (81.2)	112 (65.1)	462 (76.6)
African-American	67 (15.5)	57 (33.1)	124 (20.6)
Others	14 (3.2)	3 (1.7)	17 (2.8)
Cohort			
STRRIDE I	145 (33.6)	69 (40.1)	214 (35.5)
STRRIDE AT/RT	135 (31.3)	48 (27.9)	183 (30.3)
STRRIDE-PD	151 (35.0)	55 (32.0)	206 (34.2)
Intervention components			
Diet = 0	392 (91.0)	164 (95.3)	556 (92.2)
Diet = 1	39 (9.0)	8 (4.7)	47 (7.8)
*Resistance = 0	346 (80.3)	150 (87.2)	496 (82.3)
*Resistance = 1	85 (19.7)	22 (12.8)	107 (17.7)
Amount = 0	45 (10.4)	19 (11.0)	64 (10.6)
Amount = 1	254 (58.9)	98 (57.0)	352 (58.4)
Amount = 2	132 (30.6)	55 (32.0)	187 (31.0)
Intensity = 0	45 (10.4)	19 (11.0)	64 (10.6)
Intensity = 1	155 (36.0)	53 (30.8)	208 (34.5)
Intensity = 2	231 (53.6)	100 (58.1)	331 (54.9)

*P-values <.05 between completers and dropouts. Percentages in parentheses represent the percent within each category. *SD* standard deviation.

### Genetic SNPs Associated With Dropout

**Fig. 2.**
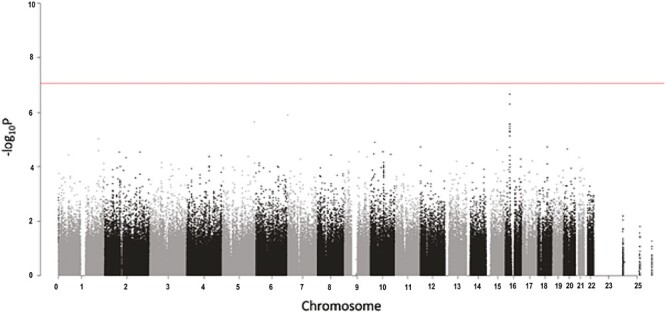
Manhattan plot of the STRRIDE dropout genome-wide association study (GWAS) results across all chromosomes with a log_10_-based *P*-value for 584,117 single-nucleotide polymorphisms (SNPs).

An overview of SNP GWAS results across all chromosomes is shown as a Manhattan plot in Fig. 2. SNP results were ranked by *p*-value. No SNP reached genome-wide significance (*p*-value, 5 × 10^−8^); there was suggestive evidence for 56 SNPs associated with dropout at *p* < 1 × 10^−4^, among those 12 SNPs showing associations at *p* < 1 × 10^−5^, including a cluster of 9 SNPs in LD on chromosome 16. In addition, three other SNPs were also associated with dropout with *p* < 1 × 10^−5^: one SNP (rs6961510) is located in the intergenic region between *ZFAND2A* (zinc finger AN1-type containing 2A) and *UNCX* (UNC homeobox) on chromosome 7; one SNP (rs4505973) is in the intron of LOC105377731 on chromosome 5; and one SNP (rs7529338) is in an intron of *ESSRG* (estrogen receptor-related receptor gamma) on chromosome 1. GWAS results and full names of the gene loci closest to these SNPs are shown in Table 2 and Supplementary Table S2. Phenome-wide association study (PheWAS) results for exercise-related traits in the GWAS Atlas identified associations (*p* < .05) with four traits for the chromosome 16 locus SNPs (for rs722069 “Strenuous sports or other exercises” *p* = .003) [35], “Number of days/week of vigorous physical activity 10+ minutes” (*p* = .008) [34] “Vigorous physical activity” (*p* = .027) [35], and “Moderate to vigorous physical activity level” (*p* = .032) [35] (Supplementary Table S3). The “Strenuous sports or other exercises” phenotype also was associated with rs6961510 (*p* = .043) and rs4505973 (*p* = .0008); no other exercise-related phenotypes were associated at a *p* < .05 with non-chromosome 16 SNPs.

**Table 2:** Single-Nucleotide Polymorphisms (SNPs) Associated With Dropout in the STRRIDE Genome-Wide Association Study (GWAS) at *p* < 10^−5^

SNP	CHR	Map^a^ position	Test allele	MAF	OR	*P*-value	Gene
RS722069	16	23495619	C	0.265	2.234	2.19E−07	GGA2: intron variant
RS428438	16	23450433	C	0.259	2.175	5.04E−07	COG7: intron variant
RS6961510	7	1181653	A	0.318	1.946	1.25E−06	Intergenic (ZFAND2A, UNCX)
RS4505973	5	173283741	C	0.099	2.755	2.25E−06	LOC105377731: intron variant
RS7187920	16	23552180	C	0.248	2.049	2.61E−06	EARS2: synonymous variant
RS9302410	16	23500051	C	0.251	2.05	2.69E−06	GGA2: intron variant
RS11642395	16	23545240	C	0.249	2.036	3.27E−06	EARS2: intron variant
RS369856	16	23463343	C	0.246	2.036	3.92E−06	GGA2: 500B downstream variant
RS1135045	16	23478390	C	0.246	2.027	4.67E−06	GGA2: missense variant
RS120962	16	23578665	G	0.248	2.008	5.11E−06	Intergenic (UBFD1, NDUFAB1)
RS11861636	16	23428676	C	0.266	1.994	7.32E−06	COG7: intron variant
RS7529338	1	216516990	G	0.263	1.987	9.19E−06	ESRRG: intron variant

*CHR* chromosome; *MAF* minor allele frequency; *NDUFAB1* NADH:Ubiquinone Oxidoreductase Subunit AB1; *OR* odds ratio; *SNP* single-nucleotide polymorphism; *UBFD1* Ubiquitin Family Domain Containing 1.

^a^Map position from human genome build = genome reference consortium human build 38 (GRCh38).

The SNP with the lowest *p*-value (odds ratio [OR] 2.23, *p* = 2.2 × 10^−7^) was rs722069 on chromosome 16. Rs722069 is located in the intron of Golgi-associated, Gamma adaptin ear-containing, ARF-binding protein 2 (*GGA2*). Individuals with one copy of the minor allele C (vs common allele T) were 2.2 times more likely to be non-completers. The other eight SNPs on chromosome 16 were in an LD block with rs722069, located in *EARS2* (glutamyl-tRNA synthetase 2, mitochondrial) and *COG7* (component of oligomeric Golgi complex 7) gene regions close to *GGA2*. We examined population stratified results in this region which showed consistent association results in European-Americans (OR = 1.86, *p* = .0009) and African-Americans (OR = 4.40, *p* = 2.8 × 10^−5^) (Supplementary Table S4). In European-Americans rs722069 is contained in a 104-kb region of strong LD (*R*^2^ = 1.0) with multiple SNPs with strong regulatory potential [36–38]; as expected there is less LD in the African-American populations with only two SNPs in strong LD with rs722069; however, of these three SNPs, only rs722069 has published evidence of regulatory potential.

**Fig. 3.**
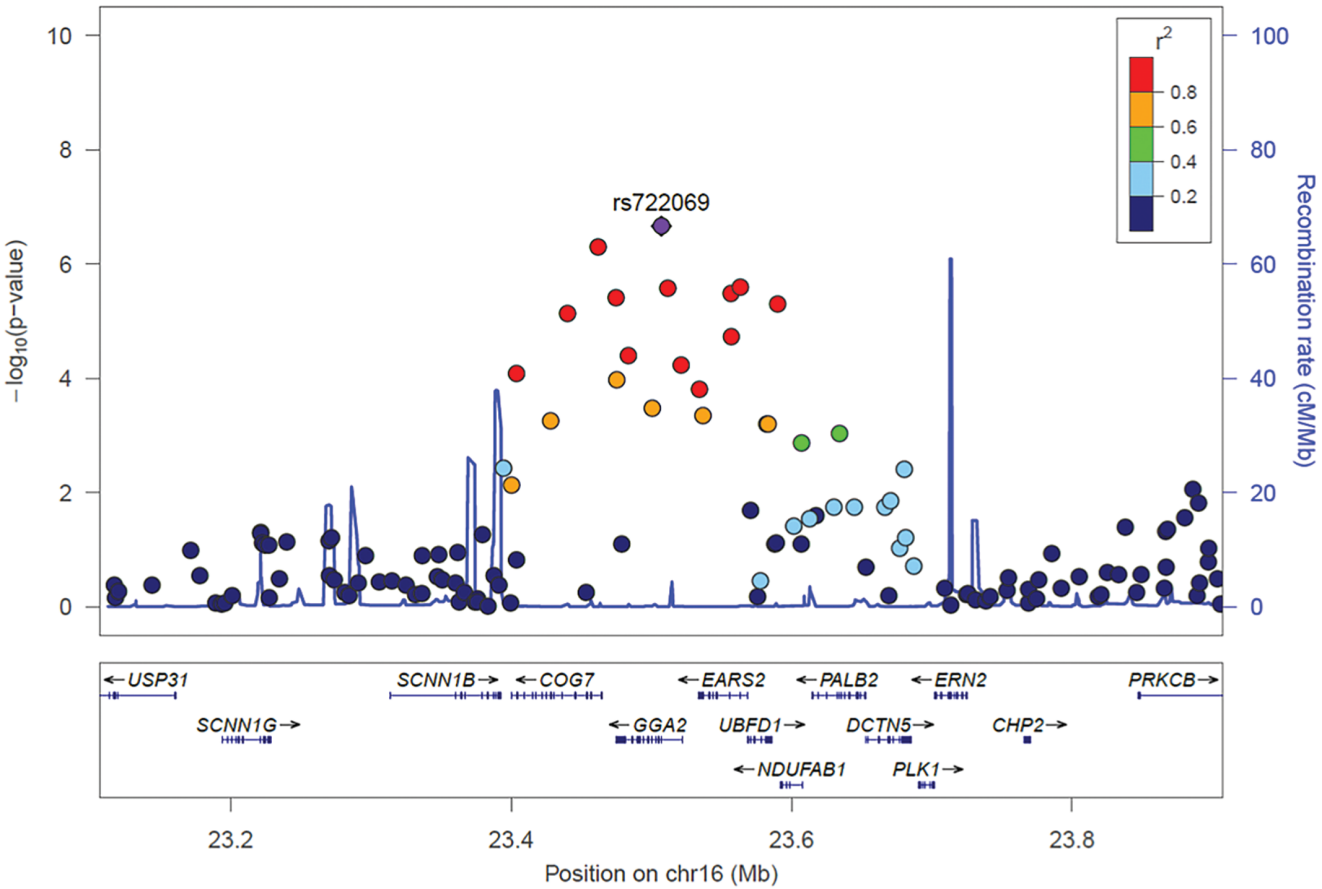
LocusZoom plot for rs722069 gene region associated with dropout in the STRRIDE genome-wide association study (GWAS).

Figure 3 shows detail of the GWAS results in the gene region containing rs722069. The SNPs and genes not on chromosome 16 demonstrated weaker support for a relationship to exercise behavior and dropout; thus, we concentrated the ‘omics analysis on the genes in the chromosome 16 region and specifically on rs722069.

### Genetic Effects on Gene Expression in Skeletal Muscle

To better understand how these variants may contribute to exercise training dropout, we hypothesized these variants may impact gene expression in untrained (before intervention) and trained (after intervention) skeletal muscle. Furthermore, to explore our hypothesis that dropout genetic candidates were associated with distinctive regulation of metabolic gene expression in skeletal muscle, we explored genes in this region of chromosome 16 for which we had evidence for genetically regulated gene expression in skeletal muscle. Using the GTEx portal, we evaluated in silico evidence for differential expression by genotype for the top SNPs in skeletal muscle. The SNP with greatest nominal effect, rs722069, is an eQTL for genes *EARS2*, *COG7*, and *DCTN5* (dynactin subunit 5), all expressed in skeletal muscle tissue. In the GTEx portal, the homozygous minor allele C/C is associated with less *EARS2* expression, greater *COG7* expression, and greater *DCTN5* expression compared to the homozygous major allele T/T (Supplementary Fig. S5).

**Fig. 4.**
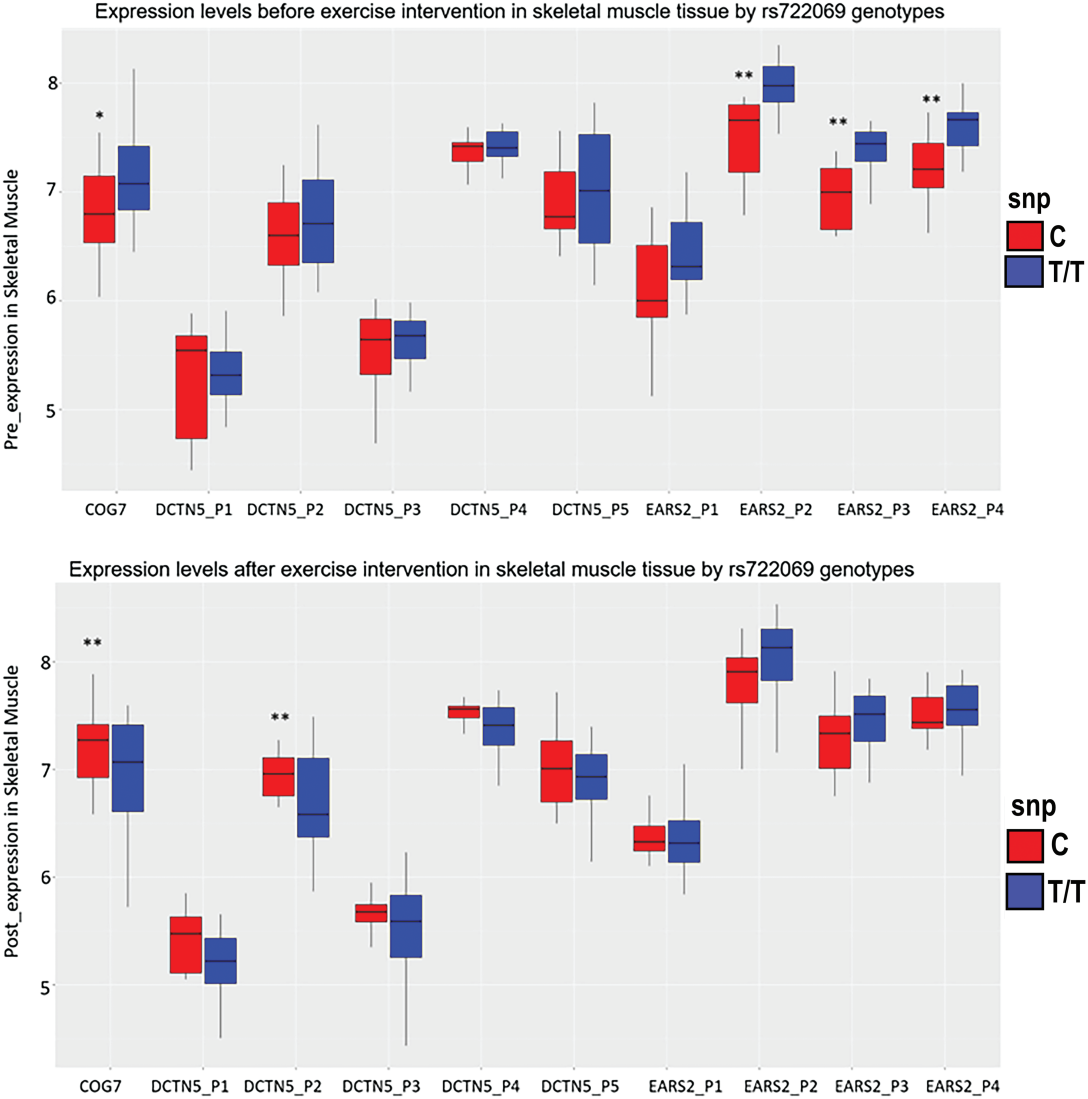
Pre- and post-intervention probe-specific expression levels in skeletal muscle for genes in the chromosome 16 region associated with dropout in the STRRIDE genome-wide association study (GWAS) by RS722069 genotypes (**P < .05, *P < .1, adjusted for age, gender, race, and study cohort, and additionally adjusted for baseline expression).

In our subset of 37 subjects with both SNP and skeletal muscle expression data available, only one subject was homozygous C/C; so, genotypes C/T and C/C were combined (C allele) for comparisons with the homozygous T/T genotype. Figure 4 shows the expression levels both before and after exercise intervention in skeletal muscle tissue with the rs722069 major T/T genotype versus the minor C allele. The minor C allele was associated with less muscle gene expression before exercise intervention (pre-expression) for the *EARS2* gene (Probes 2–4 with *p* < .0016, effect size β = −0.42 ± 0.11 to −0.5 ± 0.13;); but, it was not associated with muscle expression after exercise intervention (post-expression). Similarly, the C allele was marginally associated with less pre-expression muscle concentration on *COG7* (*p* = .074, effect size β = −0.37 ± 0.2), while the C allele was associated with greater post-expression of *COG7* (*p* = .004, β = 0.66 ± 0.21). The relationship of the C allele and greater post-expression was also present in one of five of *DCTN5* probes at Probe 2 (*p* = .024, β = 0.34 ± 0.14). In summary, there was evidence for all three of the candidate genes expressed in skeletal muscle in this genomic region—EARS2, COG7, and DCTN5—to be associated with gene expression pre-training, and limited evidence for training gene-directed effects on changes in skeletal muscle gene expression with exercise training.

### Genetic Effects on Small Molecule Metabolites in Skeletal Muscle

**Fig. 5.**
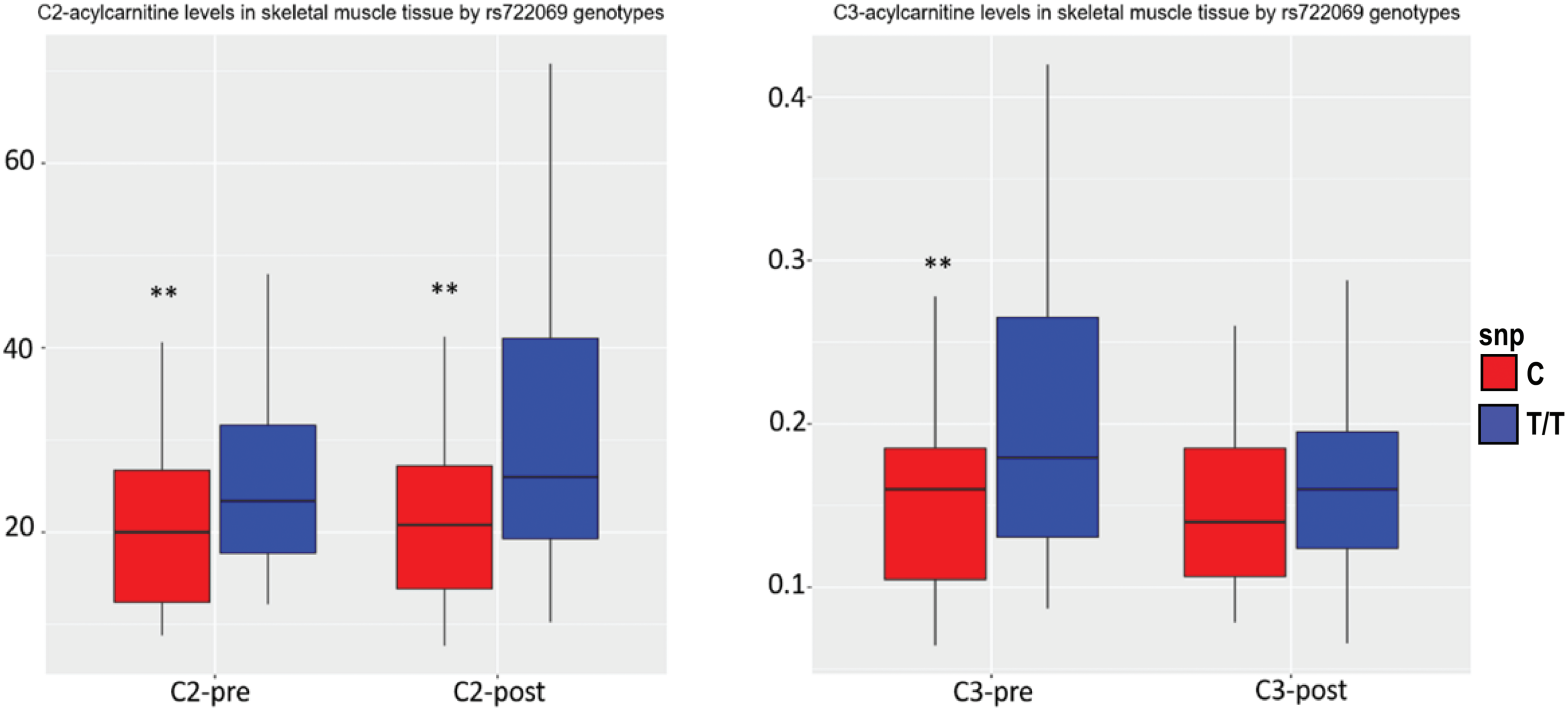
The C2- and C3-acylcarnitine concentrations in skeletal muscle by RS722069 genotypes (**P < .05, adjusted for age, gender, race, study cohort, and batch effect, and for post-training metabolites, additionally adjusted for baseline metabolite concentration). *C2-post* C2-acylcarnitine after exercise intervention; *C2-pre* C2-acylcarnitine before exercise intervention; *C3-post* C3-acylcarnitine after exercise intervention; *C3-pre* C3-acylcarnitine before exercise intervention.

To explore our hypothesis that dropout genetic variants were associated with distinctive regulation of metabolic state in skeletal muscle, we explored skeletal muscle metabolites for which we had evidence for gene regulated gene expression in skeletal muscle. To focus on the end products for even and odd number carbon length metabolic intermediates, we chose to concentrate attention on the C2 and C3 acylcarnitine’s—the C2 (acetyl; pyruvate) project being the end-product of amino acid, fatty acid, and glycolytic metabolism, and the C3 (propionyl) being the next to last product of odd carbon-chain length metabolism. These are the best-measured and most abundant acylcarnitines related to energy metabolism in skeletal muscle and represent flux through these common energy generating metabolic pathways. Figure 5 shows the C2- and C3-acylcarnitine concentrations in skeletal muscle tissue by rs722069 major T/T genotype versus the minor C allele. In the subset of available SNP and skeletal muscle metabolite data (*n* = 82), the minor C allele of rs722069 was associated with lesser pre-metabolite (*p* = .005, β = −6.48 ± 2.21) and post-metabolite concentrations (*p* = .044, β = −4.48 ± 2.19) of C2-acylcarnitine in muscle—the carnitine ester of acetyl CoA—as the end-product of beta-oxidation. The C allele was also associated with lesser pre-metabolite muscle concentrations of C3-acylcarnitines (*p* = .026, β = −0.090 ± 0.029), byproducts of branched-chain amino acid oxidation.

### Gene Expression Associated With Small Molecule Metabolites in Skeletal Muscle

In the subset of available skeletal muscle expression and metabolite data (*n* = 54), lesser pre-expression of *COG7* was associated with lesser pre-metabolite muscle C2-acylcarnitine (*p* = .009, β = 0.41 ± 0.15). Lesser *EARS2* (Probes 2 and 3) gene expression pre-training was associated with lesser muscle C3-acylcarnitine pre-training (*p* = .017, β = 0.49 ± 0.20; *p* = .024, β = 0.52 ± 0.22, respectively) (Supplementary Fig. S6). There was also a trend for lesser gene expression of *EARS2 pre-training* with lesser muscle C3-acylcarnitine pre-training (Probe 1 with *p* = .09, β = 0.29 ± 0.17; Probe 3 with *p* = .055, β = 8.09 ± 4.10). Greater pre-training expression of DCTN-5 (Probe 3 with *p* = .078, β = 0.33 ± 0.18) was marginally associated with greater post-training C3-acylcarnitine concentration.

## Discussion

Following previous work using a candidate gene approach to identify an association of metabolic genes related to exercise intervention dropout [15], we undertook a GWAS of exercise intervention dropout in our three STRRIDE studies [23–25]. Our ad hoc hypothesis was that exercise intervention dropout would be associated with muscle gene expression and metabolites related to energy metabolism. The analysis identified associations with several SNPs in LD in the region of chromosome 16p12.2 harboring the genes *COG7*, *GGA2*, *EARS*, and *DCTN5* associated with an increased likelihood of exercise intervention dropout. Further strengthening these findings were eQTL associations, where untrained persons with the C allele of rs722069 had lesser skeletal muscle gene expression of *EARS2* and fewer byproducts of fatty acid and branched-chain amino acid catabolism (C2- and C3-acylcarnitine). Additionally, independent of genotype, in the untrained state, muscle expression of both SNP-related *COG7* and *EARS2* gene expression was associated with lesser concentrations of muscle C2- and C3-acylcarnitines; lesser EARS2 expression and C2- and C3-acylcarnitine concentrations may reflect reduced mitochondrial content and/or quality [26]. These findings indicate that exercise intervention dropout is, in part, genetically moderated through alterations in gene expression and metabolic pathways in skeletal muscle—acting through impaired mitochondrial energetics and Golgi function in skeletal muscle—and indicating less energy available for muscle contraction and/or other active metabolic processes. As in our previously published work [15], the baseline impairments in peripheral muscle metabolism mediated by genetic variation and consistent alteration of molecular processes involved in muscle energy metabolism observed in this study—mitochondrial energetics and Golgi function in the skeletal muscle—may lead to greater perceived exertion and pain during exercise, subsequently de-motivating the participant, and resulting in exercise intervention dropout. Further investigation of these hypotheses should occur in prospective studies specifically designed to test them.

### Function of the Chromosome 16.2 Region

The genetic locus in the Manhattan plot most strongly identified with the genetic variant rs722069, contains seven coding genes in four functional categories. Given the strong LD in this region and strong regulatory potential across the region, it is difficult to identify a single causal variant or gene. Several of the genes in this region are putative candidates for skeletal muscle response to exercise. There are genes involved in mitochondrial function and metabolism (EARS2); Golgi-, peroxisome-dependent protein processing (COG7, GGA2); and subcellular trafficking (DCTN5).

The EARS2 gene product (glutamyl-tRNA synthetase 2) is a nuclear-encoded mitochondrial protein; a member of the class I family of aminoacyl-tRNA synthetases. The protein product is coded in the nucleus, imported into mitochondria where it catalyzes the ligation of glutamate to its cognate tRNA molecule. Genetic mutations in this gene are associated with combined oxidative phosphorylation deficiency 12 (COXPD12), an autosomal recessive mitochondrial neurologic disorder [39]. The disorder can be considered part of a family of genetically heterogeneous combined oxidative phosphorylation deficiency conditions best characterized by COXPD1 variants. In our studies, a subset of the STRRIDE sample, the C allele of our strongest SNP association with exercise intervention dropout—rs722069—was inversely associated with pre-exercise eQTL of *EARS2*. The C allele was inversely associated with pre-exercise skeletal muscle concentrations of C2- and C3-acylcarnitines—the incomplete oxidation products of fatty acid and amino acid metabolism. Lesser *EARS2* skeletal muscle gene expression also directly corresponded to lesser C2- and C3-acylcarnitine concentrations in skeletal muscle before exercise. These mitochondrial metabolic deficiencies would likely be associated with actual and perceived lack of response to exercise training.

The COG7 gene product (component of oligomeric Golgi complex 7; alias CDG2E) is a nuclear-encoded protein residing in the Golgi [40–43]. This gene codes for one of eight subunits constituting the conserved oligomeric Golgi (COG) complex, which is required for normal Golgi assembly and function. Of interest, mutant variants of this gene are associated with impaired integrity of the Golgi complex; aberrant trafficking; disruption of Golgi-dependent glycosylation pathways; and a congenital disorder—glycosylation type IIe. The rs722069 variant is an eQTL for skeletal muscle gene expression for COG7 mRNA in GTEx, which confirmed our analyses. In addition, this variant was associated with differential response of COG7 gene expression to exercise training in our studies. Furthermore, these attenuations of COG7 gene expression with exercise training were associated with attenuations of exercise training-induced skeletal muscle metabolite concentrations. Thus, a mild functional variant, such as rs722069, results in modest downregulation of gene expression for a very important constituent of Golgi function. This downregulation may be responsible for actual and perceived lack of ability to effectively train as compared to those not carrying the variant.

The DCTN5 gene product (dynactin subunit 5) codes for a dynactin protein subunit—a cofactor for the microtubule motor cytoplasmic dynein-1 [44]. The gene has eight splice variant transcripts. The rs722069 is an eQTL for DCTN5 gene expression in skeletal muscle in GTEx, which was confirmed in STRRIDE samples.

The rs722069 variant is an intronic SNP in the GGA2 gene. The GGA2 protein product is a member of the Golgi-localized, gamma adaptin ear-containing, ARF-binding (GGA) family. This protein is believed to be involved in cargo molecules regulation and clathrin-coated vesicle assembly [45–47]. Our transcriptome arrays did not have probes for the GGA2 gene; therefore, we could not draw conclusions about the potential role of this candidate gene in mediating biological effects on exercise dropout in our studies.

Given these known and unknown metabolic pathways, our underlying hypothesis for this work holds that exercise intervention dropout is genetically moderated through differential gene expression and metabolic pathway activities in skeletal muscle. Therefore, the mitochondria-associated and metabolic protein, EARS2, would be assumed to be the best functional candidates consistent with our hypothesis. More work is needed to understand how the Golgi-associated genes/proteins—COG7 and GGA2 and DCTN5 genes may influence exercise intervention dropout. The variant associated with intervention dropout in this region may act to coordinately regulate a more complex genetic program responsible for our observations.

Current research surrounding exercise intervention dropout primarily focuses on psychosocial and environmental factors [48]—such as lack of time and motivation—overlooking genetic and metabolic predispositions that influence an individual’s behavior. This study is one of the first to explore genetic and metabolic determinants as underlying factors influencing exercise intervention dropout. Although further investigation is necessary in this area of research, these findings provide molecular targets that may be important to assess prior to an individual initiating an exercise intervention. If an individual is found to have one of these at-risk alleles or differentiation in gene expression at baseline, personalized approaches might be utilized to target adoption to the exercise intervention. Future studies should combine these identified genes and metabolites associated with exercise intervention dropout, with psychosocial and environmental factors, to begin to draw a map of the behavior change process that occurs during an exercise intervention.

Strengths of the current study include being a first of its kind analysis, with the results being generalizable to the large component of the modern American population with overweight or obesity, metabolic syndrome components of elevated blood pressure, glucose intolerance, insulin insensitivity short of diabetes, and atherogenic dyslipidemia. Findings may be less generalizable to other populations. We had a relatively large cohort of well-characterized individuals who underwent a supervised exercise intervention. We assessed skeletal muscle molecular studies across three -omics platforms. Last, despite a relatively small sample size for a GWAS, we found a strong signal for loci on chromosome 16 associated with exercise intervention dropout. This signal is supported by genotype-specific expression and metabolite levels in skeletal muscle. However, this study does not come without limitations. As stated, we had a small sample size for a GWAS, as well as a very small subset of the total GWAS being analyzed for gene expression and metabolites. Given the small sample size, we were not able to do a full stratified GWAS by genetic ancestry. However, the statistical models adjusted for genetic ancestry through the use of genetic principal components and we examined the results for the top SNPs in African-Americans and European-Americans separately; the association results were similar, suggesting robustness of the top SNPs to confounding by ancestry, along with consistency of results in two independent ancestry groups. For those who dropped out from the exercise intervention, no muscle expression or metabolite data were collected. Replication and validation of this analysis will be necessary to solidify our current findings.

There are some limitations to this secondary analysis specific to the clinical exercise trial. We recognize it is difficult to ascribe the underlying motivations and reasons for dropout from an exercise intervention or program. Some reasons, such as “moved out of the region” are clearly not motivated by the program itself, but likely by other factors beyond the scope of our exercise study. These could be for family, for work reasons, or others. This occurred in eight individuals and a sensitivity analysis performed to exclude those individuals from the analysis failed to demonstrate any significant impact on our findings. Additional information regarding the sensitivity analysis can be found in the Supplementary Materials.

A more difficult situation is presented by those individuals “lost to follow-up.” It is difficult—rather, nearly impossible—to ascribe a reason for this occurrence to either biologically motivated behavior or nonbiologically motivated behavior in all instances. We can say, however, that those who are motivated to continue exercise habits once adopted do so under the most extreme circumstances, such as in extreme weather or while traveling. Such individuals are receiving significant biological and psychological reinforcement for their exercise behavior from continued participation in their exercise habits. For these reasons, we did not believe it reasonable to exclude individuals in the “lost-to-follow-up” category from the analysis. We believe these are more likely to be true biologically driven and behaviorally motivated dropouts. To fairly address these limitations will require a prospective clinical study specifically designed to address the issue of genetic predictors of exercise behavior.

## Conclusion

These findings imply exercise intervention dropout is genetically moderated through alterations in gene expression and metabolic pathways in skeletal muscle; furthermore, impaired mitochondrial energetics and Golgi function in skeletal muscle may be partly responsible for dropout behavior. Individual genetic traits may allow the development of a biomarker-based, targeted approach for identifying individuals who may benefit from more intensive counseling to adopt an exercise intervention.

## Supplementary Material

kaae011_suppl_Supplementary_Material

## Data Availability

The deidentified genotype and gene expression data supporting the conclusions of this article are available in the dbGAP repository (https://www.ncbi.nlm.nih.gov/gap/, accession number: phs001855.v1.p1) and GEO repository (https://www.ncbi.nlm.nih.gov/geo/, accession number: GSE48278). Other deidentified data are not available in a public archive. These deidentified data can be made available (as allowable according to the institutional IRB standards) through a request to Dr. William Kraus.
