# Supplementary material for: Genome-Wide Genetic Analysis of Dropout in a Controlled Exercise Intervention in Sedentary Adults With Overweight or Obesity and Cardiometabolic Disease
Source: Ann Behav Med. 2024 Mar 15;58(5):363–74. doi: 10.1093/abm/kaae011 (PMC11008589; doi:10.1093/abm/kaae011)
Supplement: kaae011_suppl_Supplementary_Material [file kaae011_suppl_supplementary_material.docx]

**Supplemental Files:**

**Exercise Training Information.**

In STRRIDE I, subjects were randomly assigned to one of four groups for center-based, supervised exercise training: (1) non-exercising control; (2) low volume/moderate-intensity aerobic exercise, defined as a caloric equivalent of 12 miles/week at 40–55% peak oxygen consumption (peak VO_2_); (3) low volume/vigorous intensity, defined as the caloric equivalent of 12 miles/week at 65–80% peak VO_2_; and (4) high volume/vigorous-intensity exercise, defined as the caloric equivalent of 20 miles/week at 65–85% peak VO_2_.

In STRRIDE AT/RT, subjects underwent a four-month control run-in period followed by an eight-month exercise intervention in one of four exercise groups for center-based, supervised exercise training: (1) low volume/vigorous intensity group, identical to the low volume/vigorous intensity group of STRRIDE I; (2) resistance training, in which subjects completed a regimen of three sessions per week during which nine resistance exercises were performed with eight to twelve repetitions at 70–85% of one repetition maximum weight; (3) low volume/vigorous-intensity aerobic exercise plus resistance training, during which subjects completed the low volume/vigorous-intensity aerobic training protocol in addition to the resistance training protocol; and (4) high volume/vigorous-intensity aerobic training, identical to that of STRRIDE I.

In STRRIDE-PD, subjects underwent a six-month center-based, supervised exercise training intervention in one of three exercise-only or one exercise plus diet group: (1) high volume/vigorous intensity group, identical to the high volume/vigorous intensity group of STRRIDE I; (2) high volume/moderate-intensity aerobic exercise; (3) low volume/moderated intensity aerobic exercise, identical to the low volume/vigorous intensity group of STRRIDE I; and (4) low volume/moderate-intensity aerobic exercise combined with a diet program designed to achieve a 7% weight loss over six months—identical to the first six month intervention period in the Diabetes Prevention Program.

**Sensitivity Analysis.**

We identified eight individuals whose dropout reason was geographic relocation, which may be less related to genes. In order to properly evaluate our analyses, we conducted a sensitivity analysis without those individuals and found the effect size and p-value are similar to the originally reported values included in the manuscript. Sensitivity analysis results: Odds ratio = 2.28, p-value = 1.7x10-7.

| **Table S1.** Digital coding of STRRIDE study exercise groups as described in the text. | | | |  |
| --- | --- | --- | --- | --- |
| **STRRIDE I Intervention Code** | | | | |
| Exercise Group | Diet | Resistance | Amount | Intensity |
| Control | 0 | 0 | 0 | 0 |
| Low-Amount/Moderate-Intensity | 0 | 0 | 1 | 1 |
| Low-Amount/Vigorous-Intensity | 0 | 0 | 1 | 2 |
| High-Amount/Vigorous-Intensity | 0 | 0 | 2 | 2 |
| **STRRIDE AT/RT Intervention Code** | | | | |
| Exercise Group | Diet | Resistance | Amount | Intensity |
| Low-Amount/Vigorous-Intensity (AT-Mod) | 0 | 0 | 1 | 2 |
| High-Amount/Vigorous-Intensity (AT-High) | 0 | 0 | 2 | 2 |
| Aerobic Training/Resistance Training | 0 | 1 | 1 | 2 |
| Resistance Training | 0 | 1 | 0 | 0 |
| **STRRIDE-PD Intervention Code** | | | | |
| Exercise Group | Diet | Resistance | Amount | Intensity |
| Low-Amount/Moderate-Intensity | 0 | 0 | 1 | 1 |
| High-Amount/Moderate-Intensity | 0 | 0 | 2 | 1 |
| High-Amount/Vigorous-Intensity | 0 | 0 | 2 | 2 |
| Low-Amount/Moderate-Intensity/Diet | 1 | 0 | 1 | 1 |

| **Table S2.** Single nucleotide polymorphisms (SNPs) associated with dropout at p < 1×10 ^-4^ in the STRRIDE genome-wide association study (GWAS) of dropout. | | | | | | | | | |
| --- | --- | --- | --- | --- | --- | --- | --- | --- | --- |
| CHR | SNP | BP | Test Allele | OR | SE | L95 | U95 | STAT | P |
| 16 | RS722069 | 23495619 | C | 2.234 | 0.1551 | 1.648 | 3.027 | 5.183 | 2.19E-07 |
| 16 | RS428438 | 23450433 | C | 2.175 | 0.1547 | 1.606 | 2.946 | 5.025 | 5.04E-07 |
| 7 | RS6961510 | 1181653 | C | 1.946 | 0.1374 | 1.487 | 2.548 | 4.848 | 1.25E-06 |
| 5 | RS4505973 | 173283741 | C | 2.755 | 0.2143 | 1.81 | 4.194 | 4.729 | 2.25E-06 |
| 16 | RS7187920 | 23552180 | C | 2.049 | 0.1527 | 1.519 | 2.764 | 4.699 | 2.61E-06 |
| 16 | RS9302410 | 23500051 | C | 2.05 | 0.153 | 1.519 | 2.767 | 4.694 | 2.69E-06 |
| 16 | RS11642395 | 23545240 | C | 2.036 | 0.1528 | 1.509 | 2.747 | 4.653 | 3.27E-06 |
| 16 | RS369856 | 23463343 | C | 2.036 | 0.1541 | 1.506 | 2.754 | 4.616 | 3.92E-06 |
| 16 | RS1135045 | 23478390 | C | 2.027 | 0.1543 | 1.498 | 2.743 | 4.579 | 4.67E-06 |
| 16 | RS120962 | 23578665 | G | 2.008 | 0.1529 | 1.488 | 2.71 | 4.56 | 5.11E-06 |
| 16 | RS11861636 | 23428676 | C | 1.994 | 0.1539 | 1.475 | 2.696 | 4.484 | 7.32E-06 |
| 1 | RS7529338 | 216516990 | G | 1.987 | 0.1548 | 1.467 | 2.691 | 4.435 | 9.19E-06 |
| 10 | RS2505502 | 25699598 | A | 2.083 | 0.1679 | 1.498 | 2.894 | 4.368 | 1.25E-05 |
| 12 | RS2107523 | 1547195 | G | 0.5175 | 0.1537 | 0.3829 | 0.6994 | -4.286 | 1.82E-05 |
| 18 | RS11082830 | 50564902 | A | 1.804 | 0.1377 | 1.378 | 2.364 | 4.286 | 1.82E-05 |
| 16 | RS11643602 | 23545588 | G | 1.93 | 0.1538 | 1.428 | 2.609 | 4.275 | 1.91E-05 |
| 20 | RS808682 | 19735958 | C | 1.852 | 0.1455 | 1.393 | 2.464 | 4.236 | 2.28E-05 |
| 15 | RS2292713 | 58173284 | C | 0.3471 | 0.2511 | 0.2122 | 0.5679 | -4.213 | 2.52E-05 |
| 1 | RS12745466 | 216523585 | A | 1.901 | 0.1527 | 1.409 | 2.564 | 4.205 | 2.61E-05 |
| 9 | RS990000 | 77471618 | C | 2.049 | 0.1714 | 1.464 | 2.867 | 4.185 | 2.86E-05 |
| 10 | RS2894081 | 69295476 | T | 1.775 | 0.1372 | 1.357 | 2.323 | 4.184 | 2.87E-05 |
| 2 | RS6710164 | 79073389 | T | 0.4655 | 0.1833 | 0.325 | 0.6667 | -4.172 | 3.01E-05 |
| 2 | RS16832254 | 190024535 | A | 0.5021 | 0.1651 | 0.3633 | 0.694 | -4.172 | 3.02E-05 |
| 10 | RS379242 | 19832227 | A | 1.806 | 0.1428 | 1.365 | 2.389 | 4.14 | 3.47E-05 |
| 10 | RS11194725 | 109571062 | A | 1.852 | 0.1491 | 1.382 | 2.481 | 4.131 | 3.61E-05 |
| 1 | RS689258 | 54664069 | C | 1.853 | 0.1497 | 1.382 | 2.485 | 4.119 | 3.81E-05 |
| 8 | RS1107217 | 72824531 | T | 1.741 | 0.1347 | 1.337 | 2.267 | 4.119 | 3.81E-05 |
| 4 | RS2280665 | 185264139 | G | 0.5384 | 0.1507 | 0.4007 | 0.7234 | -4.109 | 3.98E-05 |
| 16 | RS7204714 | 23472032 | C | 2.007 | 0.1699 | 1.439 | 2.801 | 4.101 | 4.12E-05 |
| 5 | RS1864255 | 140594888 | T | 1.745 | 0.136 | 1.336 | 2.277 | 4.092 | 4.27E-05 |
| 4 | RS7695341 | 120121057 | A | 0.5528 | 0.1449 | 0.4162 | 0.7344 | -4.091 | 4.30E-05 |
| 9 | RS10985563 | 122203423 | C | 0.5597 | 0.1423 | 0.4235 | 0.7397 | -4.08 | 4.51E-05 |
| 2 | RS17043407 | 114715944 | T | 0.5257 | 0.158 | 0.3857 | 0.7165 | -4.07 | 4.70E-05 |
| 21 | RS1735901 | 20439669 | A | 1.749 | 0.1375 | 1.336 | 2.29 | 4.065 | 4.80E-05 |
| 7 | RS1485693 | 68990513 | A | 1.838 | 0.1507 | 1.368 | 2.469 | 4.038 | 5.39E-05 |
| 16 | RS8056064 | 82753448 | G | 2.066 | 0.1798 | 1.453 | 2.94 | 4.036 | 5.43E-05 |
| 16 | RS727238 | 23509745 | C | 1.889 | 0.1584 | 1.385 | 2.577 | 4.014 | 5.97E-05 |
| 17 | RS7222848 | 54851242 | G | 2.575 | 0.2364 | 1.62 | 4.093 | 4.002 | 6.29E-05 |
| 13 | RS9598442 | 62366916 | A | 1.812 | 0.1488 | 1.354 | 2.425 | 3.994 | 6.49E-05 |
| 9 | RS11795390 | 24805163 | G | 1.935 | 0.1655 | 1.399 | 2.676 | 3.989 | 6.63E-05 |
| 15 | RS3817428 | 88872016 | G | 1.873 | 0.1576 | 1.375 | 2.551 | 3.982 | 6.84E-05 |
| 10 | RS4148946 | 72010315 | C | 0.5525 | 0.1492 | 0.4124 | 0.7402 | -3.976 | 7.00E-05 |
| 3 | RS4688398 | 63450793 | C | 0.5682 | 0.1424 | 0.4298 | 0.7512 | -3.969 | 7.22E-05 |
| 6 | RS11967409 | 18115484 | T | 2.242 | 0.204 | 1.503 | 3.345 | 3.959 | 7.52E-05 |
| 5 | RS4868506 | 175211213 | A | 1.756 | 0.1422 | 1.329 | 2.32 | 3.958 | 7.55E-05 |
| 14 | RS965680 | 33102024 | C | 0.4833 | 0.1838 | 0.3372 | 0.6929 | -3.957 | 7.61E-05 |
| 1 | RS10754842 | 244639403 | T | 0.5299 | 0.1608 | 0.3866 | 0.7262 | -3.95 | 7.82E-05 |
| 18 | RS11082824 | 50463828 | C | 1.772 | 0.1449 | 1.334 | 2.353 | 3.947 | 7.91E-05 |
| 16 | RS250583 | 23392189 | A | 1.828 | 0.1533 | 1.354 | 2.469 | 3.935 | 8.32E-05 |
| 3 | RS3796249 | 114025421 | A | 2.21 | 0.2017 | 1.488 | 3.282 | 3.932 | 8.43E-05 |
| 10 | RS896076 | 72015949 | G | 1.763 | 0.1446 | 1.328 | 2.34 | 3.921 | 8.81E-05 |
| 12 | RS1012093 | 1546618 | G | 0.5573 | 0.1493 | 0.4159 | 0.7468 | -3.915 | 9.04E-05 |
| 6 | RS6909725 | 18117137 | G | 2.19 | 0.2002 | 1.479 | 3.242 | 3.915 | 9.04E-05 |
| 4 | RS2715990 | 120247607 | C | 0.4757 | 0.1907 | 0.3273 | 0.6912 | -3.897 | 9.74E-05 |
| 13 | RS9604529 | 113919624 | G | 1.861 | 0.1593 | 1.361 | 2.543 | 3.896 | 9.77E-05 |
| 10 | RS1245584 | 72017185 | A | 1.757 | 0.1448 | 1.323 | 2.334 | 3.895 | 9.81E-05 |

| **Table S3.** Genome-wide association study (GWAS) Atlas results for seven exercise intensity and amount traits for the top single nucleotide polymorphisms (SNPs) shown in Table 2**.** | | | | | | | | | | | | |
| --- | --- | --- | --- | --- | --- | --- | --- | --- | --- | --- | --- | --- |
| **Trait** | **RS**  **722069** | **RS**  **428438** | **RS**  **6961510** | **RS**  **4505973** | **RS**  **7187920** | **RS**  **9302410** | **RS**  **11642395** | **RS**  **369856** | **RS**  **1135045** | **RS**  **120962** | **RS**  **11861636** | **RS**  **7529338** |
| Number of days/week walked 10+ minutes | 0.008 | 0.009 |  |  | 0.008 | 0.009 | 0.009 | 0.009 | 0.009 | 0.011 | 0.011 | - |
| Number of days/week of moderate physical activity | - | - | - | - | - | - | - | - | - | - | - | - |
| Number of days/week of vigorous physical activity | - | - | - | - | - | - | - | - | - | - | - | - |
| Strenuous sports or other exercises | 0.003 | 0.005 | 0.043 | 0.0008 | 0.003 | 0.003 | 0.003 | 0.004 | 0.002 | 0.003 | 0.016 | - |
| Vigorous physical activity | 0.027 | 0.044 | - | - | 0.028 | - | 0.032 | 0.038 | 0.034 | 0.035 | 0.038 | - |
| Moderate to vigorous physical activity level | 0.032 | 0.027 | - | - | 0.022 | - | 0.024 | 0.022 | 0.022 | 0.022 | 0.012 | - |
| Types of physical activity in last 4 weeks: Strenuous sports | - | - | - | - | - | - | - | - | - | - | - | - |
| Total phenotypes <.05 | 176 | 175 | 184 | 163 | 171 | 155 | 137 | 163 | 155 | 207 | 175 | 237 |
| Total phenotypes <.01 | 36 | 31 | 56 | 43 | 34 | 31 | 30 | 29 | 25 | 34 | 38 | 76 |

| **Table S4.** Ancestry-stratified association results with exercise dropout for single nucleotide polymorphism (SNP) rs722069 in the STRRIDE exercise intervention trials**.** | | | | | | | | | |
| --- | --- | --- | --- | --- | --- | --- | --- | --- | --- |
| Ancestry | SNP | Effect Allele | EAF | N | OR | SE | L95 | U95 | p-value |
| African-American | RS722069 | C | 0.504 | 125 | 4.4 | 0.3597 | 2.17 | 8.9 | 3.83E-05 |
| European-American | RS722069 | C | 0.198 | 462 | 1.86 | 0.1882 | 1.29 | 2.7 | 0.000933 |
| All | RS722069 | C | 0.265 | 603 | 2.23 | 0.1551 | 1.65 | 3.03 | 2.19E-07 |
| Abbreviations: SNP = single nucleotide polymorphisms, EAF = effect allele frequency; n = number of participants; OR = odds ratio; SE = standard error; L95 = lower endpoint of the confidence interval; U95 = upper endpoint of the confidence interval. | | | | | | | | | |

**
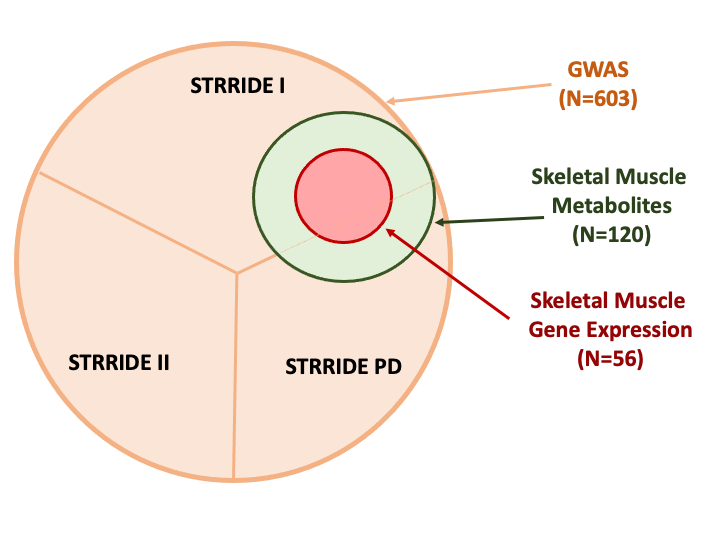
**

**Figure S1. Sample sizes for STRRIDE intervention trials, genome-wide association study (GWAS), and genomic analyses.** The GWAS for STRRIDE included 603 participants completing exercise training in the three contributing studies. Of this population. 120 across five exercise groups and one control group had metabolites in skeletal muscle measured before and after exercise and 60 had skeletal muscle transcriptome analysis.

**
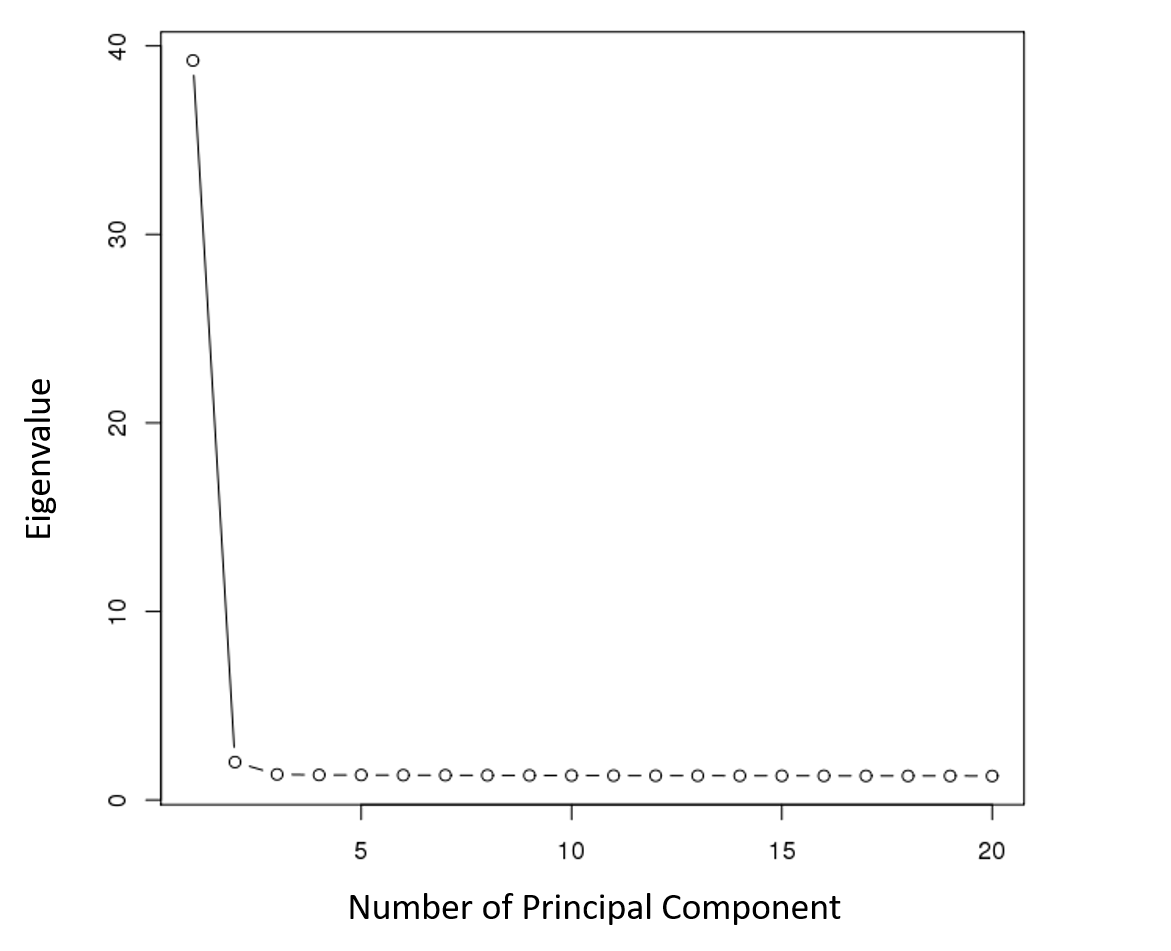
**

**Figure S2:** Scree plot of the top 20 principal components for the STRRIDE genome-wide association study (GWAS) of dropout.

**
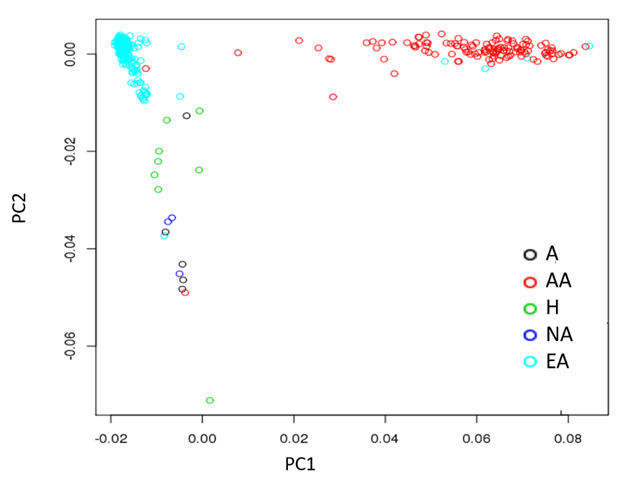
**

**Figure S3.** Scatter plot of 1^st^ and 2^nd^ Principle components by self-reported ancestry among 603 samples (A-Asian, AA-African American, H-Hispanic, NA-Native American, EA-Caucasian).


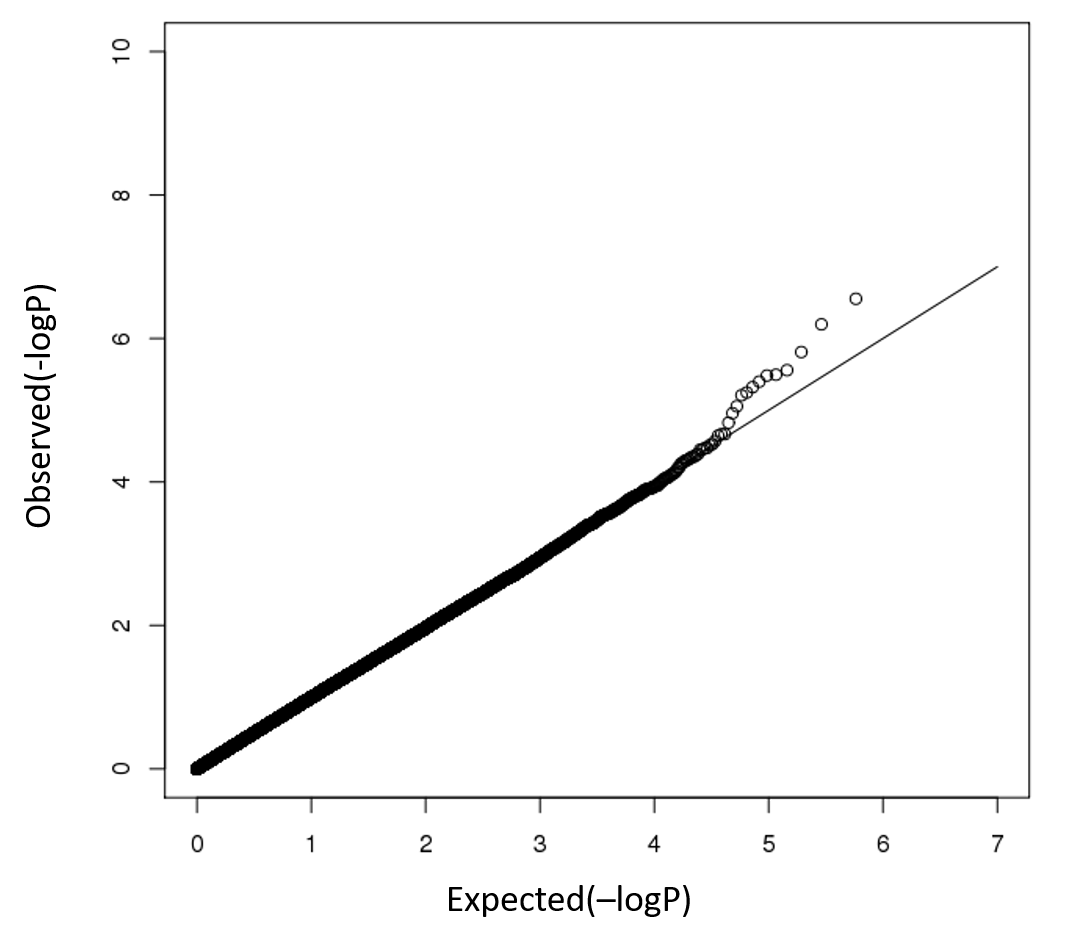


**Figure S4.** Q-Q plot of single nucleotide polymorphisms (SNPs) associated with dropout in 603 STRRIDE samples.

**
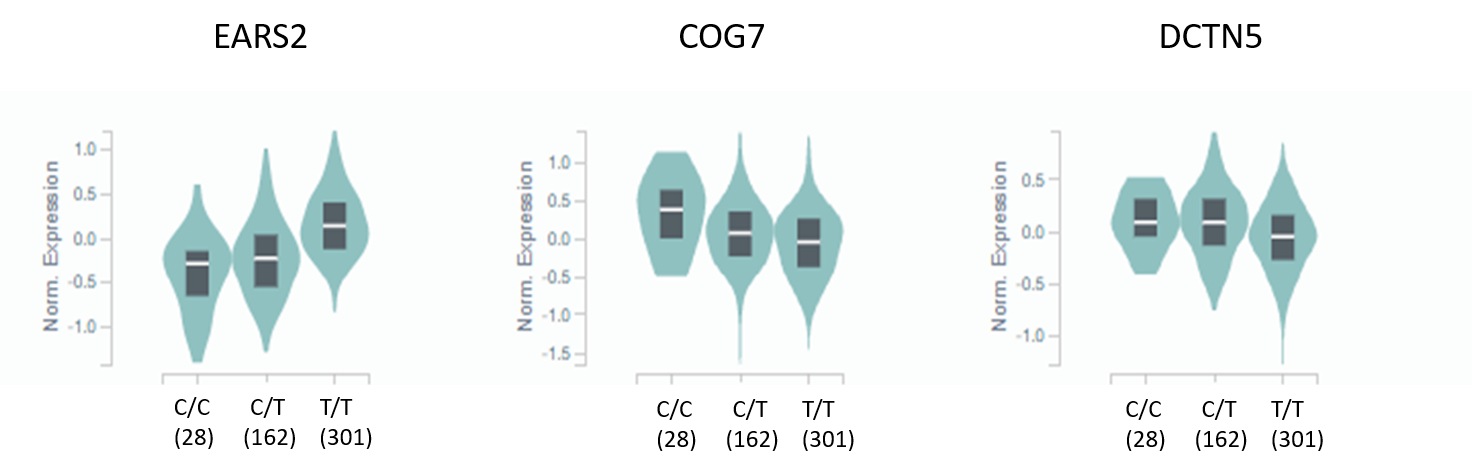
**

**Figure S5.** Expression quantitative trait loci (eQTL) violin plots of RS722069 in skeletal muscle (from Genotype-Tissue Expression (GTEx) portal).

**
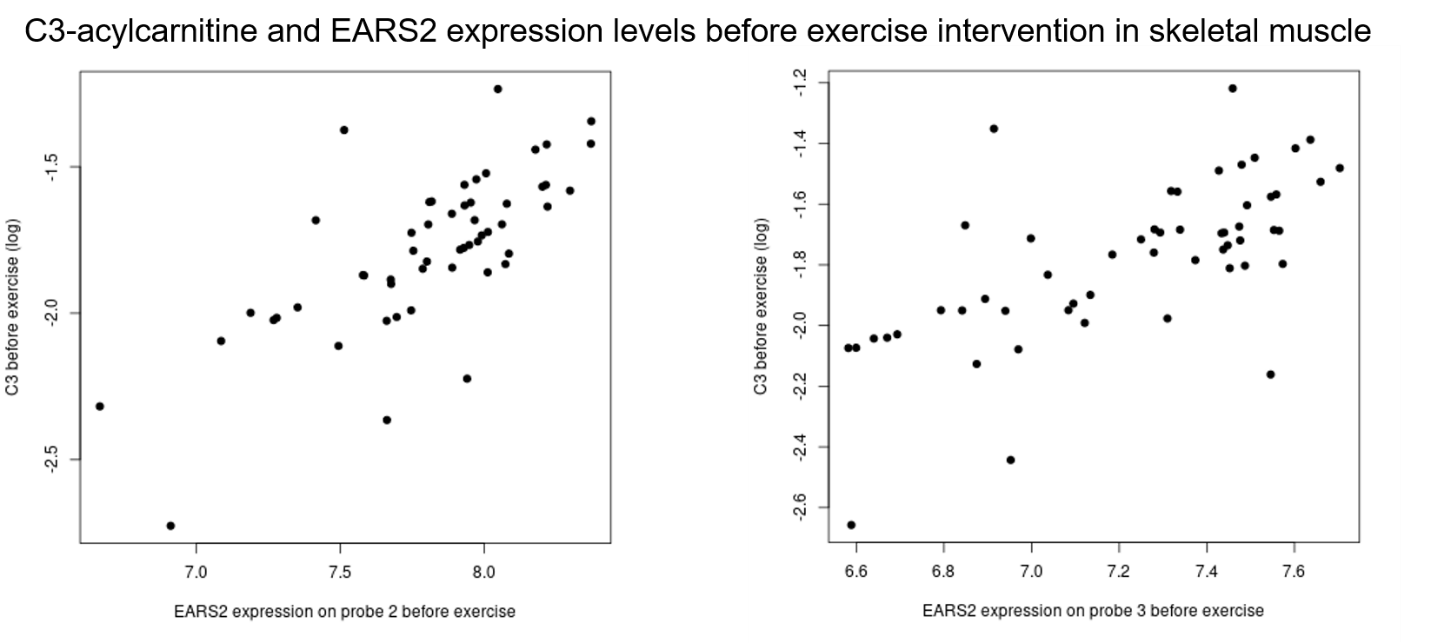
Figure S6.** Scatter plots of C3-acylcarnitine and glutamyl-tRNA synthetase 2 (EARS2) expression levels (Probe 2 AND probe 3) in skeletal muscle before the exercise intervention.
